# Exploring a Gaming-Based Intervention for Unemployed Young Adults: Thematic Analysis

**DOI:** 10.2196/44423

**Published:** 2024-01-18

**Authors:** Ingjerd Jevnaker Straand, Asbjørn Følstad, Jone Ravndal Bjørnestad

**Affiliations:** 1 Department of Social Work University of Stavanger Stavanger Norway; 2 Department of Sustainable Communication Technologies SINTEF Digital Oslo Norway

**Keywords:** positive psychology intervention, digital mental health, serious gaming, intervention design, research through design, gaming-based intervention

## Abstract

**Background:**

Promoting positive psychologies that promote resilience such as a growth mindset could be beneficial for young, unemployed adults, as many lack the self-esteem and self-efficacy to cope with job search adversity. These young people may be reached at scale through the web-based delivery of self-administered positive psychology interventions. However, past studies report unsatisfying user experiences and a lack of user engagement. A gaming-based experience could be an approach to overcoming these challenges.

**Objective:**

Our research objective was to explore how young, unemployed adults experience a positive psychology intervention designed as a game to extract learning and principles for future intervention research and development.

**Methods:**

To respond to the research question, a team of researchers at the University of Stavanger worked with designers and developers to conceptualize and build a gaming-based intervention. Feedback from the users was collected through formative usability testing with 18 young adults in the target group. Retrospectively, recordings and notes were transcribed and subjected to thematic analysis to extract learnings for the purposes of this paper.

**Results:**

A total of 3 themes were identified that pinpoint what we consider to be key priorities for future gaming interventions for unemployed young adults: adaptation to user preferences (eg, need for responding to user preferences), empathic player interaction (eg, need for responsiveness to user inputs and a diverse set of interaction modes), and sensemaking of experience and context (eg, need for explicit presentation of game objectives and need for management of user expectations related to genre).

**Conclusions:**

Feedback from end users in usability-testing sessions was vital to understanding user preferences and needs, as well as to inform ongoing intervention design and development. Our study also shows that game design could make interventions more entertaining and engaging but may distort the intervention if the game narrative is not properly aligned with the intervention intent and objectives. By contrast, a lack of adaptation to user needs may cause a less motivating user experience. Thus, we propose a structured approach to promote alignment between user preferences and needs, intervention objectives, and gameplay.

## Introduction

### Positive Psychology for Unemployed Young Adults

Young people who are not in education, employment, or training (NEET) comprise, on average, 12.8% aged between 15 and 29 years in the Organisation for Economic Cooperation and Development countries [1]. Studies show that negative self-perceptions and a lack of perseverance are barriers to successful labor market inclusion [2-4], as the new labor market requires highly skilled workers who are not afraid of change, challenges, and acquiring new skills [5,6]. For young people with weak beliefs in their capacity to learn, this could be a major risk factor for labor market exclusion, and this may in turn impact their overall well-being. Several researchers have studied the relationship between unemployment and mental health. McGee and Thompson [7] found a relationship between unemployment and depression in young adults and suggested the use of psychological interventions for the young and unemployed. The Norwegian NEET group is more likely to be recipients of health-related benefits, have poorer mental health, and lower levels of education compared with the average of the Organisation for Economic Cooperation and Development [8,9]. A qualitative inquiry into young people’s own experience of unemployment in Norway points to poor self-efficacy and lack of self-esteem that are reinforced through challenges and setbacks, even when these initially occur beyond the individual’s control [10,11], such as when there are insufficient training placements on offer for the vocational school pupils, a problem leading to a relatively large number of unqualified school dropouts in Norway [9]. Thus, there is a substantial rationale for exploring further how the public can offer training, not only in job-seeking skills, such as curriculum vitae (CV) writing and gaining work skills, but also in building psychological well-being and resilience to cope with such setbacks and challenges [12,13].

In the context of a broader research project, the Career Learning App, our study investigates the design and development of a web-based intervention using positive psychology to achieve beneficial changes [14]. Our broader research idea is that young people in the NEET group, henceforth referred to as *young, unemployed adults* (for the sake of simplicity and to reduce stigma), could benefit from building confidence in the possibility of learning and improving. The research idea stems from a body of work that has demonstrated positive results from offering high school students self-administered positive psychology interventions (PPIs) centered on growth mindset and challenge-seeking behaviors [15,16]. A growth mindset is the belief that human capacities are not fixed but can be developed and increased in response to one’s own efforts, good strategies, and help from others [17]. If a simple web-based PPI can influence high schoolers’ mindsets in ways that lead to positive academic outcomes [15,16,18-20], then it could also likely be beneficial to the young unemployed, leading to changes in how they engage with their contexts. Despite this strong rationale for the applicability of PPIs to facilitate well-being and personal growth in vulnerable populations, they have only, to a limited extent, been tested and used in the context of unemployment [13,21]. However, we cannot simply apply the PPIs designed for educational contexts; they need substantial adaptation to be relevant or usable for this new target group of young, unemployed adults [17]. For instance, the school-related examples used within the PPI to make them relatable are not relevant to this new target population. Furthermore, there is a lack of shared context to piggyback on to deliver the intervention and ensure that users will adhere to it. Thus, there is a need to design and develop a web-based PPI designed specifically for young, unemployed adults and their context. If successful in user studies, a resulting intervention may be used in forthcoming large-scale randomized controlled trials in Norway.

### Problems With Self-Administered Interventions

**Figure 1:**
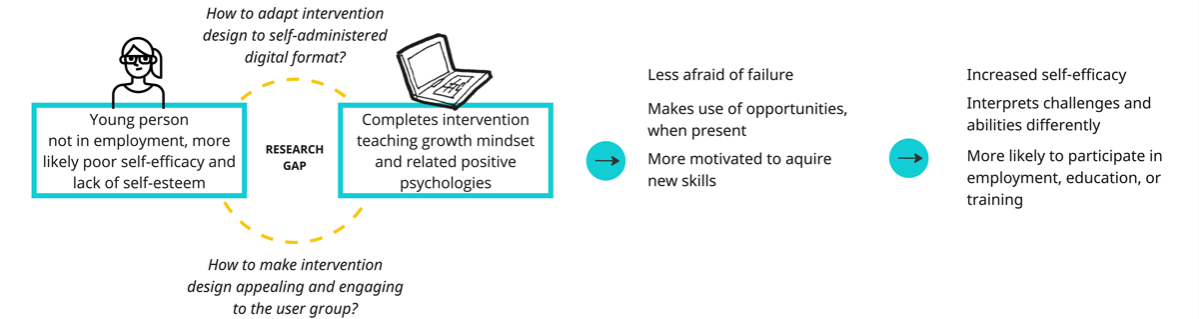
How mindset change may positively impact young, unemployed adults and the challenges of adherence, which we see as related to a research gap with a lack of knowledge of intervention designing.

Self-administered web-based interventions have the potential to support well-being and positive health changes in a large number of people at a moderate cost [22]. However, this introduces new challenges, illustrated in Figure 1. First, there is the challenge of adapting current PPIs to self-administered digital formats that are fit for the purpose of the intended user population. Second, there is the challenge of user motivation to obtain the users to complete and adhere to the intervention [23,24]. Past research suggests that users are not interested in or do not enjoy using digital mental health interventions [25], suggesting a need to work on the actual interventions themselves to increase engagement and user motivation.

### Exploring PPI as Gameplay to Be Relevant for Young People

Past research suggests a need to adapt to the media preferences of young people and make the apps more visual and interactive to increase engagement and motivation among young people [26]. One possible approach to increasing engagement is to explore games and game elements. Starting from “where the young people are at” makes pedagogical sense [27], thus the application of game design is founded on young people’s own interests as a way to foster engagement and learning of positive psychologies. Although play and games are not unique to young humans [28,29], the average age of video game players is now 33 [30]. However, playing video games continues to be popular among young people [30,31]. Interactive digital games are increasingly used for purposes beyond entertainment, as exemplified by the rise of health gaming apps for video gaming consoles. Game design elements are also increasingly applied to nongame contexts, for instance, by adding points and badges to nongame experiences, such as social media networks [32,33] or learning contexts [34]. When game design and game concepts are being applied for purposes beyond fun, they may be termed “serious games,” “learning games,” or “gamification” [35-40]. Game design applied to learning may be seen as a form of experiential learning (eg, learning-by-doing) [41,42]. Game design has been successfully applied to mental health interventions [43,44] and educational contexts [34,38] in the past. Game design offers an approach to creating engaging experiences. Engagement is a complex and ambiguous term [45]. Our use of the term is in the sense of “emotional involvement” as in offering a pleasurable experience [32] and to describe how motivational, usable, and acceptable [46] the game would be in the eyes of the target audience, because this could be an important predictor of adherence. In general, there is insufficient research on the application of gaming and gamification to mental health, particularly in the well-being domain [47], and we have not found empirical studies that pursue to gamify positive psychology targeted specifically toward unemployed young people. There were no available gaming-based PPIs that could be used for the purposes of this study.

### Research Objective

**Figure 2:**
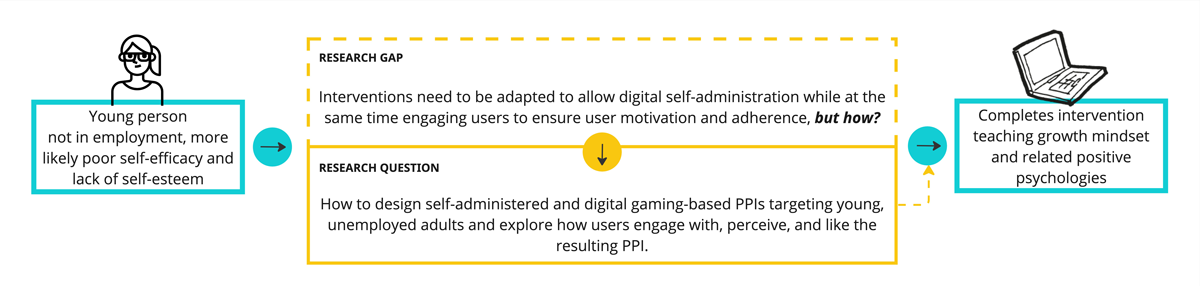
How the research question is linked with the research gap identified. PPI: positive psychology intervention.

Our limited knowledge of how to adapt the intervention from an educational setting to a game-based format suggested a highly explorative approach, where we identified the need to design a game to explore this topic and to overcome the gaps in knowledge summarized in Figure 2. Thus, the research objective and question of this study were how to design a self-administered and digital PPI in a gaming format targeting young, unemployed adults and to explore how they engage with the game and whether they like using it.

## Methods

### Setting: Learning in “Action”

**Figure 3:**
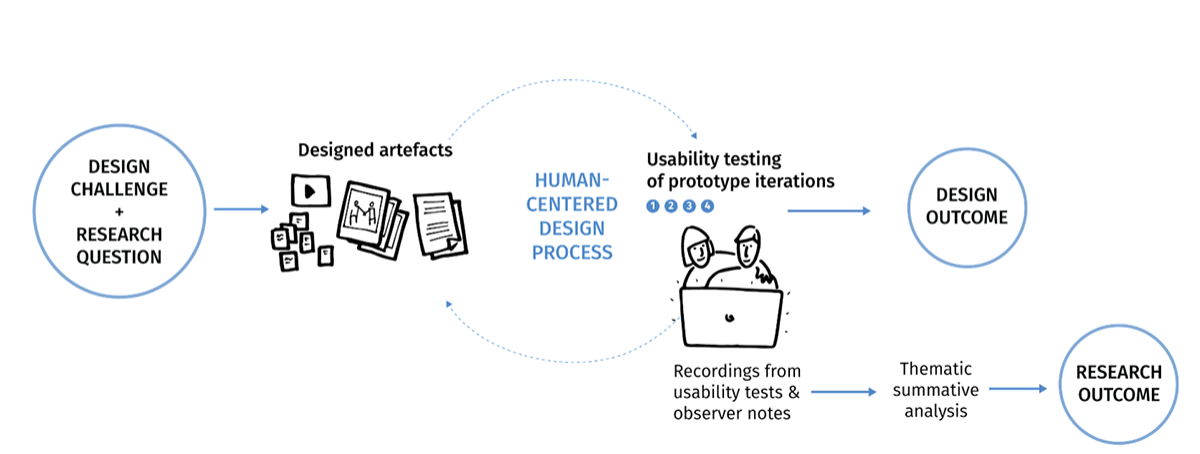
A Research through Design process with design outcomes and research outcomes.

To answer our research question, this study used a human-centered design process [48,49], an approach that allows input from target users during design and development. The human-centered design process is a form of research with design as the primary outcome [50]. The collection of feedback from users during design and development impacts not only the design of the game but also the production of knowledge. As such, it is a form of participatory action-research [51,52] where “doing research” and “doing action” happen simultaneously. In the design research literature, this may also be referred to as “Research through Design” [53,54], where knowledge is produced through the design of the artifact and through the experience of the artifact. At the end of the project, we analyzed user feedback data thematically [55] to extract the learnings and design principles for the potential application toward creating user-friendly and user-relevant gaming-based PPIs for vulnerable populations. Figure 3 summarizes the “Research through Design” approach of this study.

### Data Collection Through User Testing

We used testing with users to capture the user experience. Testing with users is important as a tool for obtaining the “design right” in game design [56]. Our project-specific usability study [57] was formative, and we were qualitatively evaluating early prototypes to answer how and why questions as a means to improve the design [58,59]. We aimed to capture participants’ *thinking*, for example, opinions, reasoning, and attitudes toward the prototype experience. The various prototypes thus functioned as exploratory “hypothesis testing” [60,61] and as boundary objects [62-64] that framed conversations with end users. These sessions with the end users lasted from 45 to 90 minutes, where users were asked to briefly describe their background and interests, followed by open “think-aloud” questions [65] related to a prototype experience, such as “What do you think about what you see here?” “What do you expect will happen now?” and “What do you think this is?” Thus, the participants were encouraged to verbalize their thoughts and experiences. Afterward, the participants were asked follow-up questions that were equally open, such as “What are you thinking now that you have seen this?” and ‘How would you describe this to a friend?” The objectives of usability testing were to collect feedback related to broad aspects of the intervention experience, namely its (1) engagement, (2) relatability, (3) understandability, and (4) potential for improvement.

### Sample and Recruitment

The NEET group includes anyone not in economic activity from the age of 15 to 29 years [1]. The European Foundation for the Improvement of Living and Working Conditions has defined 7 subgroups of NEETs [66], ranging from the “classically unemployed” to young people who are caretakers, unable to work, or simply listed as “inactive.” How long an individual remains a NEET also varies significantly. The diversity of the population is not necessarily problematic for our study; when designing with users, one would usually strive for variance [49,67] rather than representativeness. Our study is exploratory and does not require large samples [68]. Our estimate required 20 users; however, this sample size was highly approximate, in line with qualitative studies in general [69,70]. Pragmatic needs in the design process guided the number of participants to a large degree and not, for instance, theoretical saturation.

We recruited from the Norwegian Labour and Welfare Administration (NAV), the national welfare institution of Norway that pays out unemployment wages and social support, and from a regional Individual Placement and Support (IPS) program, which offers placement support to young people with first-episode psychosis. We also recruited through the user testing platform, Teston (UserTesting). Our inclusion criteria were an age range of 18 to 29 years, with a “NEET background,” and because of the language in the game prototype, living in Norway and speaking Norwegian. We say “NEET background” and not “NEET status” because our participants from the IPS program were no longer in the NEET group by definition. We made a deliberate choice not to exclude based on the length of NEET status and unemployment. Although those who are entering the NEET group in the short term, the “in-betweeners” [66], often find new employment without assistance [71] relatively quickly, even a short time out-of-work may increase the risk of exclusion [72]. We did not recruit participants on permanent disability allowance. Using multiple channels enabled quicker recruitment during the COVID-19 pandemic and increased the variance in our sample (as desired), as young people without the rights to receive unemployment benefits have fewer incentives to register with NAV [73]. We did not compare the experience of the intervention based on the recruitment channel because the groups were overlapping and experienced varying prototypes depending on the stages in the design process.

### Participants

In total, 18 participants (12/18, 67% females, 6/18, 33% males) took part in the study during the 21 testing sessions; thus, some participants were involved more than once. Recruitment was particularly challenging because of the COVID-19 pandemic, and we found that it was difficult to recruit young, unemployed men. Remote participation through web-based technologies, such as the Zoom (Zoom Video Communications) platform, enabled the study to continue during the lockdown. We also experienced that this user group continued to prefer remote participation, even when restrictions were lifted. Table 1 summarizes the participant statistics and format of the usability test.

**Table 1:** Age distribution of participants and format of user testing.

Recruitment channel	Format user testing	Age (years)^a^
IPS^b^	In person+Remote	19
IPS	In person+Remote	28
IPS	In person+Remote	18
IPS	In person+Remote	27
IPS	In person	22
IPS	Remote	19
IPS	Remote	18
IPS	Remote	23
Teston	Remote	18-29
Teston	Remote	18-29
Teston	Remote	18-23
NAV^c^	Remote	21
NAV	Remote	18
NAV	In person	21
NAV	Remote	21
NAV	Remote	18
NAV	Remote	18
NAV	In person	22

^a^For 3 (17%) of the 18 participants, we only had an age interval provided to us.

^b^IPS: Individual Placement and Support.

^c^NAV: Norwegian Labour and Welfare Administration.

### The Design and Development Process

**Figure 4:**
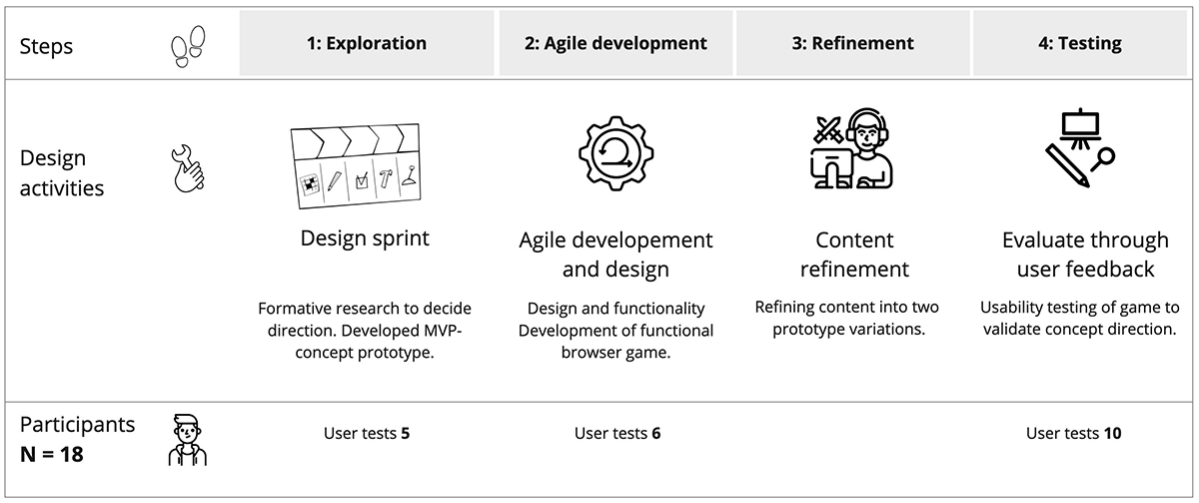
The stepwise design and development process.

Researchers at the University of Stavanger worked with designers and developers from a consulting company and potential future end users in an agile [60] and human-centered design [48,49] process. The objective of the process was to create an enjoyable gaming-based PPI targeting unemployed young adults. This process took place in 4 steps over approximately 9 months, from 2019 to 2020. In the first step, (1) *design exploration* in the form of a design sprint, a 5-day design and prototyping process [61], produced a minimum viable product of a gaming concept that was tested with 5 participants. Following a brief period for planning and procurement, we moved on to (2) *agile development*, consisting of 3 sprints, each lasting about a month. During this step, usability tests were conducted on 6 participants. This was followed by (3) *refinement* of content and prototypes, with involvement from behavioral intervention researchers to further develop and “add-in” the necessary intervention content. Finally, the prototypes were evaluated using (4) *testing*. In this step, 10 participants participated in usability testing and provided feedback on the final set of prototypes. Figure 4 summarizes the stepwise design and research process.

The intervention content needed to be adapted to be meaningful and relatable to the user group [74]. The basis for our gaming-based PPIs were growth mindset interventions from the “National Study Learning Mindset” [16] and its translated Norwegian version, “U-SAY” [5,15]. These are interventions that target high school students [15,16]. We added selected parts of cognitive behavioral therapy [75], specifically management of negative emotions, panic, and anxiety, to offer a more productive interpretation of stressors [76] that may occur during job search adversity [77,78]. During the first few days of the initial design sprint, a gaming concept, VitaNova, was developed where players can build a “new life” in a fictional narrative.

### The Gaming-Based Intervention

The VitaNova gaming concept is a one-player fantasy game where the user plays a “no name,” an android character that can take on any skin to morph into another character with their skills and abilities. In particular, the player can choose between being Noomi and Twizzlesprock. However, as we learn through the game, your character has a backstory as the male character Abel, a former engineer and an outcast who sees himself as a failure. The game is designed in 3D and split into episodes (missions). The game starts with very little information and instructions, landing the user right into action. This was a design tactic to spark curiosity and make the users intrigued by the game so that they would want to explore it further. As a player, your first task is to escape from captivity, and then, gradually, more and more information is being revealed to you as the game progresses. Refer to Textbox 1 for an overview of the game narrative.

**Figure 5:**
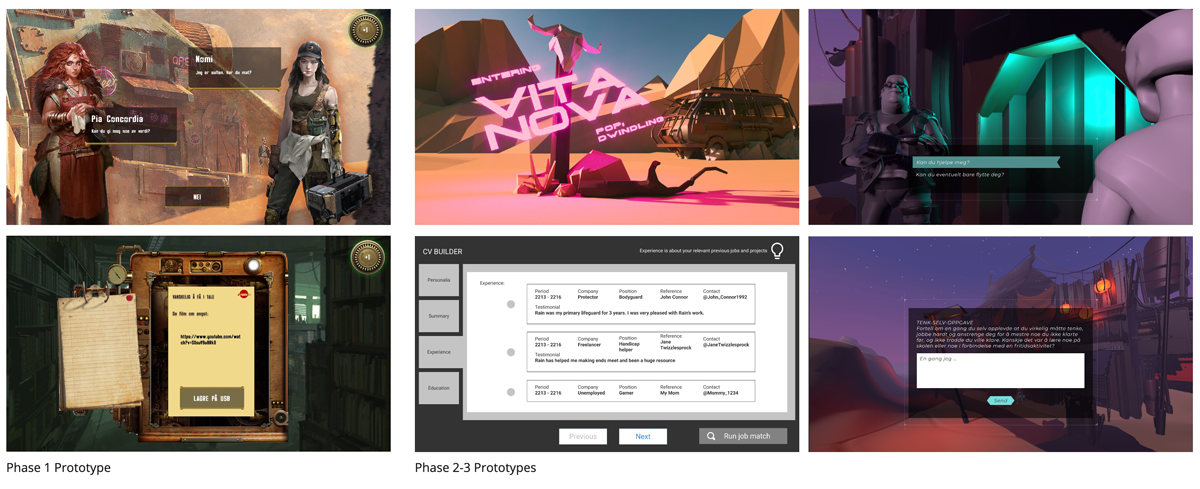
Prototype iterations of VitaNova.

The visual design of games can influence how motivational and acceptable they are to the target population [46]. Therefore, to make the game look polished, cool, and professional and to keep users immersed and engaged [79], emphasis was placed on 3D design and detailing. The game was divided into episodes, or missions. The first 3 episodes were developed into a nearly fully functional game in Unity WebGL, a platform for building 3D games that can be used in a web browser. During user testing, we also showed the prototypes and the wireframes that were made in Figma. Figure 5 shows the prototype iterations of the game design.

Psychological content and tasks are entered into the gameplay to foster psychological well-being, teach a growth mindset, and offer psychoeducation and mental health tips. Some of this is interwoven into action in the form of interactive quizzes, dialogues, or other forms of interaction, such as a CV builder applied within the game. This was intended to be transferable to the end user situation to increase relevance, although the acquisition of such practical skills was not a target of the intervention. Furthermore, there was also psychological content that was external to the gameplay, such as embedded videos. When using externally sourced content, this was implemented in the game as “ruins from the past,” which the player could “find” in the game. The player would need to watch this content and use the information provided to complete the challenges and the in-game quizzes. Upon completing an episode of the game, the player was requested to write an answer to a reflective question where the user should answer as himself or herself, to encourage internalization of the messages that had been taught in the intervention through self-persuasion [80], and to transfer learning to the user’s own situation. We have included a further description of the prototypes in Multimedia Appendix 1.

**Description of the game narrative. (The 2 final missions were not included in the user testing.)**

**1. Introduction**

Wake up in the trunk of a moving vehicle. Use hacking skills to hack the lock. Find a tavern and interact with a bodyguard who refuses your entry as “no name.” Find the Noomi skin and power unit and turn them on to enter the tavern.

**2. Tavern**

Interact with Griff and Mia at the tavern to learn about this world. Try to receive help. Tell them that you must go to an old public office to pick up energy bars if you are going to receive any help.

**3. Learn**

Find an abandoned public office where, among other things, you will discover many pieces of ancient psychological knowledge as ruins from the past.

**4. The sidekick**

Use newly acquired knowledge to help out the depressed and anxiety-ridden droid Griff, who now becomes your sidekick, and help Mia the bartender.

**5. The bully**

A bounty hunter is out looking for you to receive a reward from the boss at BetterJu Janus. Threatens your new friends at the tavern. Why are they after you?

**6. The chance**

You discover that the only way to obtain all the answers is to try to join into BetterJu. Your friends tell you of a job posting that is open. Interactive curriculum vitae and job application process.

**7. The job interview**

The job requires a different set of skills, which you acquire through entering the skin of Twizzlesprock. Job interview at the company BetterYou as Twizzlesprock.

**8. The escape**

Discover who you really are from the overhearing conversation between your new boss, Janus, and a droid. Find your ex-girlfriend, who has been trapped but confirms your true identity. You both escape from the evil boss. The end.

### Analysis

The purpose of the usability testing was to synthesize findings that led to improvements and changes in the designed outcome. Retrospectively, we also conducted thematic analysis with the steps from Braun and Clark [55,81] as a practical guide: (1) data familiarization, (2) initial code generation, (3) search for themes, (4) review themes, (5) define and name themes, and (6) produce reports [81]. All 21 usability-testing sessions were recorded. The 7 most comprehensive usability tests were transcribed verbatim. The remaining data were analyzed based on recordings, researcher notes, and memos. Specifically, we used the transcribed data as our starting point and went back to recordings and memos to review codes and themes. The analytic process was iterative and creative, where we often moved back and forth between the data and codes [82]. All authors independently familiarized themselves with the data. Author 1 started with coding using the qualitative analysis software ATLAS.ti (ATLAS.ti Scientific Software). As a group, we discussed the findings and the initial codes in a workshop before moving over to paper-based coding and printing quotes from participants organized on large paper sheets. We used diagramming, both digitally in Miro and in pen-and-paper sketches, to iterate themes and review codes in consensus meetings. Findings and concepts were discussed with other researchers, some of whom had acted as observers for the user testing or had watched recorded sessions. The quality of the analysis was ensured by researcher reflexivity, end user involvement, and method triangulation. *Researcher reflexivity* concerns activities that consider how researchers might have informed the research or biased outcomes [69]. Reflexivity was enabled through critical discussion of assumptions, themes, and codes in the team of researchers. The team also involved researchers not involved in the user testing or in the design process as an approach to validate the analysis based on these methods. *End user involvement* was supported through the iterative design process, where participant perspectives were sought at different levels of concept and design maturity. Specifically, we found it valuable to involve users from different recruitment sources (IPS, Teston, and NAV) to strengthen the credibility and transferability of the findings. *Method triangulation* was conducted by applying different approaches to design and user involvement at different phases of the process, allowing the assessment of themes or constructs from different perspectives. In particular, including data from the different phases of exploration, design, and evaluation was found to strengthen the credibility of the findings.

### Ethical Considerations

All participants provided explicit and written consent to participate in the study and were rewarded with gift cards (approximately US $30/session) for their participation. The study was evaluated and approved by the Norwegian Centre for Research Data (approval number 131074) and the regional committees for medical and health research ethics in Norway (approval number 42128).

## Results

### Overview

**Figure 6:**
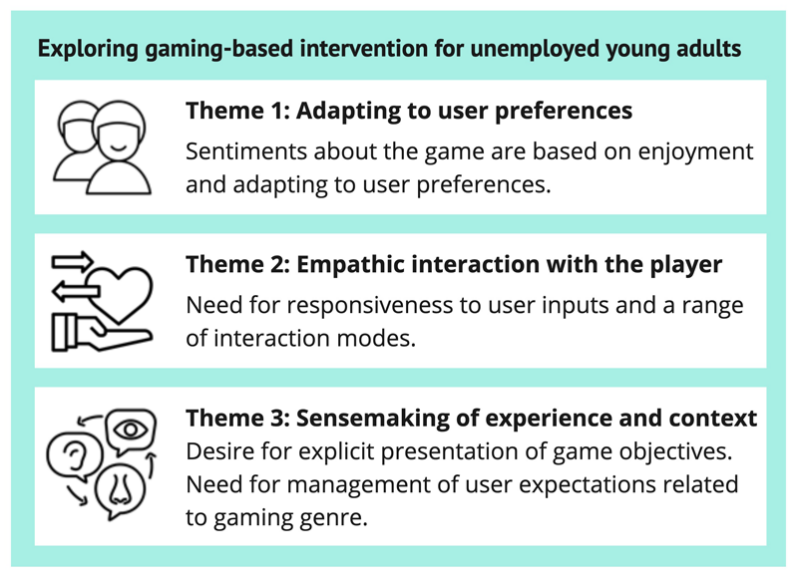
Themes and theme characteristics.

As a tool to motivate and help young adults engage in work or education, the idea of an interactive and digital game was regarded by all participants as a “good idea”; it was described as “cool,” “unexpected,” and “motivating” upon first impression. Upon closer experience with the different gaming prototypes, we received different and more specific feedback. A total of 3 themes were constructed by the researchers through active analytic engagement with the data [55]. The themes pinpoint what we consider key priorities for future gaming interventions for unemployed young adults: (1) adapting to user preferences, (2) empathic player interaction, and (3) sensemaking of experience and context. Refer to Figure 6 for an overview of the themes and their key characteristics.

### Theme 1: Adapting to User Preferences

Sentiments about the game are based on enjoyment of the game experience, the genre, and whether the game meets user preferences, either by being targeted toward them or by allowing for experience customization. The participants expressed positive feelings and excitement related to the game. It was described as much more of an *actual game* than was expected:

I liked it; it was unexpected.

P3

Seems like a cool concept. Never heard of it before.

P17

The participants pointed to the use of humor and compared the game to pure entertainment media, such as commercially available games, or other parts of popular culture, such as films or television series:

It was actually pretty exciting [laughs]. Kind of funny, considering that you have included the welfare administration here.

P6

The story was funny, it seemed a bit like a video game.

P10

The game concept was further described as “something to do” or “something to allow the time to fly,” as pure entertainment or for relaxation purposes. The relaxing features were described as something that could make one more receptive:

If it’s like that you get new assignments once a week, then it can seem exciting. At least that is something to do.

P7

So you are pretty relaxed when you get these questions, so then it is probably a little easier to answer... a little easier to reflect over this.

P4

Others felt that the gameplay story could motivate them to do something by creating a sense of urgency, where the story would drive them to make more effort. A participant wanted proof within the game that it would be worth the effort to perform mundane tasks, such as updating their CV:

That’s how it is in life too: You have to make an effort yourself to get ahead; when you have to do something here [...], then you have to hurry, because someone is after you. Then you get the adrenaline to do it.

P16

If there’s something in the game that can prove to me that it’s worth it, like writing a resume is worth it....

P18

However, several participants expressed uncertainty about whether the game would be “something for them,” suggesting either other preferences or not quite the right conceptual fit. In particular, the concept likely needed “aging up,” as it was perceived as something for a younger population. Furthermore, we learned from the participants, who spent more time gaming, that they had started out playing “adventure games” and “role-playing games” when they were younger, but that they had since moved on to playing “first-person shooter” games or other kinds of games:

It’s not a game I would have bought in the store.

P15

I think if I had been younger then, yes. Because I have a lot of different types of games that I like to play, and now I like to play games where you shoot people, but earlier I liked playing games like that, where you follow a story, for example, it’s very different in... it’s very different for people what kind of games they like to play.

P2

Designing for mobile phone use was considered important and was brought up as an improvement suggestion by nearly all users. Participants expressed that mobile phone use would make it easier to meet their own user preferences or the user preferences that they expected other young people to have:

I would have chosen an app or a mobile game. Or a course, if it was on [a] mobile [phone].

P7

Not everyone has a PC with them everywhere, so I wondered if this was on mobile.

P17

If the game could be turned into a mobile game, that would be better.

P1

Adapting to user preferences could also mean designing a customizable or more personalized experience. In particular, the choice of characters in the game is usually an arena for customization and personalization. One user commented that she would prefer to customize a character by selecting hair length, and body shape, etc rather than choosing between predefined female and male characters:

if you can choose male or female or... you don’t have to have these two, but you can choose what that person looks like. Since now a lot of young people... there are some young people who don’t want to be a man or a woman, so I think you... it’s very smart to make something like ‘do you want long hair’ ‘do you want short hair’ ‘do you want...’ [laughs]

P2

Others mentioned different strategies for choosing characters by either choosing a character that resembled themselves or identifying with them. Others would deliberately choose the extreme opposite of themselves. As one participant mentioned, “if your choice was the big male character, then you were likely ‘more vulnerable on the inside’ and ‘in need of protection.’”

### Theme 2: Empathic Interaction With the Player

This theme describes the need for responsiveness to user inputs and the desire for a range of interaction modes. Although initially intrigued and enthused by the game design, users quickly became disappointed by the lack of functionality. Thus, this theme is based on the need for empathic interaction with the player in the game, where the game needs to take the user seriously by being responsive to user inputs, thereby allowing for actual contribution to the experience:

It’s very... you can see very easily that your answers don’t make much of a difference. It doesn't matter what you choose. And I think if you’re going to have a game like this, you have to have a little change in what you say, how will it affect the game.

P2

No, it’s just that I want to see that the people you’re talking to have something else or something more to say. That they have other reactions than just “no [laughs].”

P2

It was, it’s not a challenging game, it’s not a difficult game... the game itself [laughs]. It’s designed to be clicked through. You don’t need to spend a week on it.

P3

Participants find the graphics visually appealing, although they do not feel that this is the most important aspect, saying that how the game works and how exciting and entertaining the game is are the most important parts:

I think it looks pretty nice. There are many different types of games that can be in like... many different types of ‘art’ [styles] and so, yes, there are many games that can look one way, but can still be really good, but some games that look really nice, can be boring. So, don’t worry about how the game looks, but how the game works, that’s very important.

P2

Visually it was very nice... The story I am more unsure of....

P12

Some of the feedback indicates that our game was perhaps not fully developed as a game with the necessary combination of rules, goals, feedback, fantasy, and fun [32]. Participants wish for a wider range of interaction modes, such as moving more freely around in the game, having more challenges and tasks in the game, and having different ways of interacting with the characters. Users expect game-like interactions, not just choosing answers, reading, and writing:

It is unusual for me that you cannot move around [in the game].

P4

But I think that it would be a bit boring if it was just like that you had to read, and then click to choose answer options. [...] if I was sitting at home, and this was something I had to go through every week[...] then I’d just click quickly through it. And then I hadn’t properly read what it said.

P7

To support the learning objectives of the game, players had to answer reflective questions at the end of each gaming session, where they answered as themselves and not as the gaming character. This felt a bit “off” to the participants. Furthermore, several participants expressed a problem with articulating answers to those kinds of questions, expressing that they would not know what to write when asked:

I didn’t expect that an assignment came up where you have to write about an experience from reality, sort of, which seems a little unusual to me since you are sitting inside this alternate world. But I had probably only written something about skateboarding. But it was very unusual for it to be like that.

P4

I don’t know... at least I struggle a lot with tasks like that[...] I probably wouldn’t have written anything here.

P5

Although not as exciting as hoped for, the challenges and tasks in the game can still provide the user with a sense of achievement and act as an awakening for new thoughts; if not for them, then perhaps for someone else:

You kind of get a little more confidence in yourself then. That you have actually managed something.

P6

...after all, it raises thoughts and yes... new ways of looking at things, I think. that it can start something in someone.

P7

### Theme 3: Sensemaking of Experience and Context

This theme comprises a desire for an explicit presentation of the game objectives and a need to manage user expectations related to the gaming genre. The game was described as “cool but confusing:”

Uh, well, it seems kind of cool, but it was a little hard to understand, I felt.

P5

Many participants mentioned *sensemaking* or lack of understanding in some form or another; they struggled to understand the point, the objective, or the mission to be completed in the game. Some participants pointed to a lack of logic or strangeness in the storyline and over-the-top reactions to what they perceived as minor happenings:

So people want to buy parts of dead people so they can look how they want? Hum. That’s a very strange concept! [laughs].

P2

She is stabbed now! ... And the taverna is burning. That was over-the-top. She just came for some food. This is over-the-top.

P12

The intervention messages in the game were not perceived by any of the participants. They were uncertain about what they were learning from the game experience. They were focusing on the details of the game narrative, trying to make sense of that, and, thus, the intervention part seemed “part of the fiction” and not clear what this was meant for:

Didn’t learn anything. Well... I learned that there can be different ways to solve things, but I didn’t really learn anything[...] It was a bit difficult to understand the whole story, that is the whole thing.

P1

Lots of talk about the brain, that the brain is a muscle, but don’t know what it can help with, it doesn’t make sense.

P2

The gameplay added complexity and was confusing to the participants. For most participants, there was a desire for an explicit presentation of game objectives, both in terms of what it should ultimately achieve for the end user (as an intervention) and what the objectives in the game narrative are. However, other participants felt that this uncertainty was part of the excitement:

...I should have known a little more what the goal was and what the meaning behind the game was. Because it seemed a bit like that, yes... a bit out of the blue. And you didn’t quite know what an anonymous person was and whether this was the future or whether this was a completely different world.

P7

It was very interesting. It was very unusual for me with that kind of game. But I liked how it was. And you didn’t have very much information about what you were doing so you kind of had to find out a bit about the skins and such. And I liked that.

P4

One user expressed explicit concern about how relatable the contents could be if you use a context that is far removed from everyday life:

If it becomes too sci-fi, I think it might be difficult to transfer to reality.

P18

Furthermore, we also identified a need to manage gaming genre expectations. The participants expressed a preference for certain genres over others; it may be difficult to cater to different preferences in terms of what games they like best to play. There are also certain expectations connected with different gaming genres that we were not so aware of in the research team, where users were trying to make sense of the game prototypes *in relation to* established genres, with expectations of gaming interaction to be similar to games in that genre. The participants asked us about the game in relation to genre concepts such as “open world,” “adventure,” and “role-playing” games:

So, I have a question, is this an open-world type of game where you go out to different places and pick up things or is this a text where you just follow what happens in the story?

P2

I have a question: Is this open world—or just to follow a track, like?

P17

It seems that the game genre was not clear to the participants, who pointed to different features of the gameplay prototypes that would take the game in different genre directions.

## Discussion

### Principal Findings

This study has used an iterative design process with active participation from potential users to develop an interactive game that aims to be user-friendly and engaging to be able to provide a vulnerable population with positive psychologies. As pointed out by past research, there is a strong rationale for promoting psychological well-being, for instance, to improve resilience [83] in the face of setbacks and challenges that occur as part of job search and being “out-of-work” [12,13,78], and may thus alleviate suffering [84]. A total of 3 themes were constructed from the user-based research that occurred through the design process of the gaming-based intervention: (1) adapting to user preferences, (2) empathic player interaction, and (3) sensemaking of experience and context. In the following section, we discuss the themes and how they could potentially be applied as designing principles for future self-administered gamified PPIs. Thus, the study sheds light on the application of game design for PPIs that aim to promote well-being and increase challenge-seeking in young, unemployed adults.

### Comparisons With Previous Work

#### Adapting to User Preferences

This study expands the knowledge found in other studies on PPIs for young people, where the need to offer interactive, visual, and more engaging experiences has been identified [26,85,86]. Most participants expressed uncertainty about whether the game would be “something for them,” suggesting other preferences or not quite the right conceptual fit with their preferences. We interpret this to mean that there is a need to consider gaming genres and user preferences specifically, where a more refined user segmentation may be necessary [87,88]. For instance, in our group, some participants said that they only had an interest in certain kinds of games. Future studies could consider a more fine-grained targeting strategy based on preferences and interests and not simply age and employment status. A possibility would be to segment the population based on player types [89,90] or motivation [89,91-93], and to think more carefully about user preferences for different gaming genres before choosing a concept. In our study, we found that the selected game genre was perceived as engaging “not to them,” but “someone younger.” This was particularly true for the active gamers, who found the genre to be immature. Furthermore, most participants stated that they would have preferred a game designed for mobile use, indicating another kind of context of use than our initially planned use on PCs at home.

#### Empathic Player Interaction

Inside the game experience itself, the participants in our study were disappointed by the lack of features, functionalities, and opportunities to influence what was going to happen in the game, for example, player autonomy. A lack of autonomy may cause a more negative interpretation of an experience [94]. We interpret this as underdelivery, partly because of the overpromise of the first impression and the esthetics of the graphics [95,96]. The participants expressed bleakly that in-game actions “do not matter” because, as players, they experienced an insufficient influence on the string of events in the game. Autonomy is an important motivator in self-determination theory (SDT) [97] and a lack thereof may contribute to reduced user motivation [13,35,97]. In part, the lack of autonomy and interactivity was caused by the requirements for a structured intervention set by the broader research project; each player needed to experience the same sequence of events. However, even within this frame, the game should be built to cater for somewhat more variation and focus on the interaction between the game and the player to meet user expectations. There is also a more specific need to be aware that within this target group of young, unemployed adults, many may feel *in general* that what they do does not matter [98]. Gameplay with insufficient df may unintentionally reinforce that message.

#### Sensemaking of Experience and Context

**Figure 7:**
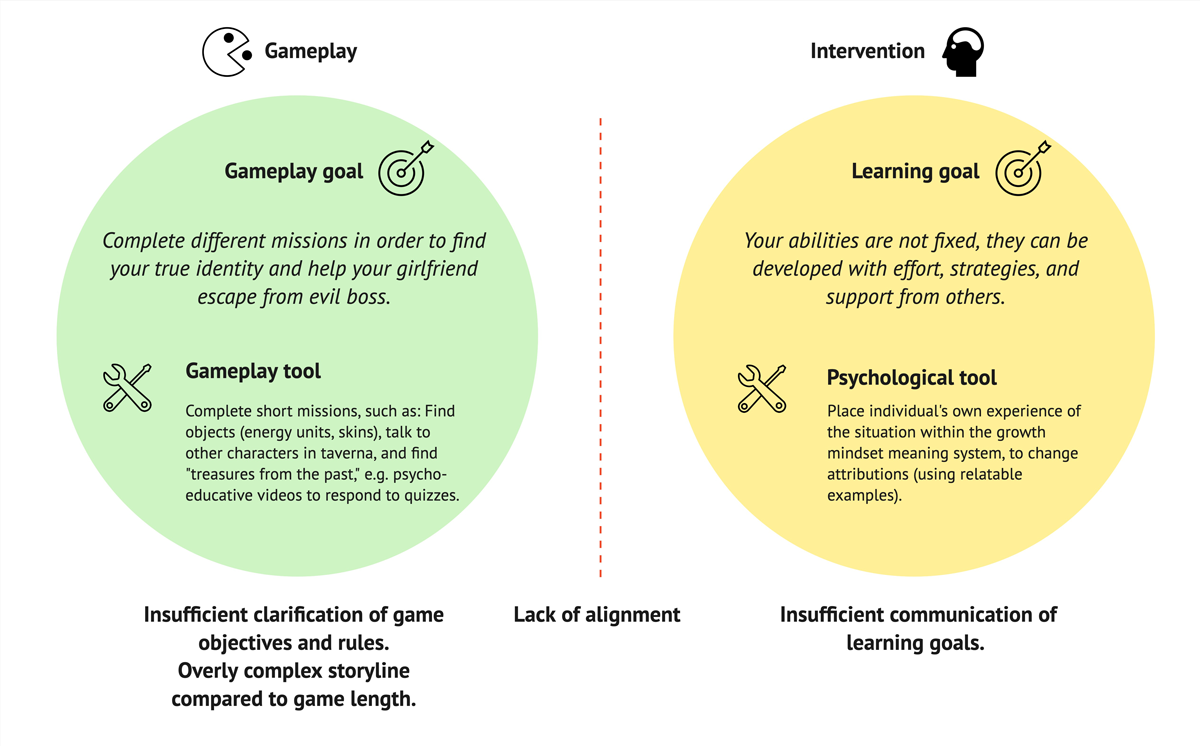
Mismatch of goals between intervention and gameplay, in combination with insufficient clarity overall.

Participants had trouble making sense of the experience: our prototypes did not meet the participants’ expectations for genre, not fitting with role-playing games based on narratives and dialogues or open-world type games, where you move around freely in a 3D world to pick up items and battle with other characters. Furthermore, the game objective and rules were either not clearly presented to the participants or did not cater to sufficient player-game dialogue and manipulation of the experience. We found that there were tensions between the gameplay and the messages of the intervention, which could undermine the intervention and potentially threaten its effectiveness. This finding is depicted in Figure 7. The PPI gameplay had a complex storyline, which confused the participants and made them miss out on their learning objectives. Past studies have pointed to psychological affordances and the importance of a “fertile soil” to make positive psychological interventions more likely to work [99-101]. In instructional or serious games used in education, Young et al [38] concluded the need to ensure that game objectives and learning objectives correspond and, further, that an overly complex gameplay can lead to misunderstandings and interfere with understanding. This seems transferable to gaming-based interventions. Other authors have referred to this as “relevant narrative,” which states that the narrative of the game should be relevant to the subject matter [102]. The choice of gameplay as a strategy for creating engagement for an intervention introduces a new context, which becomes the background for interpreting the messages of the intervention. The game design concept should be selected carefully and tested early with inexpensive methods, such as roleplay or paper sketches [32,56], to explore whether the gameplay is supportive of the intervention. In VitaNova, the gameplay goals implicitly reflected the learning goals, as the development of abilities was presented through the completion of in-game missions. However, because these learning goals were not explicitly communicated, the effectiveness of the intervention depended on the users themselves seeing the connection and transferring this knowledge to their own situation. Combined with the lack of clarity of game objectives and rules as well as an overly complex storyline, this led to confusion.

### Practical Implications for Future Gaming-Based Interventions

**Figure 8:**
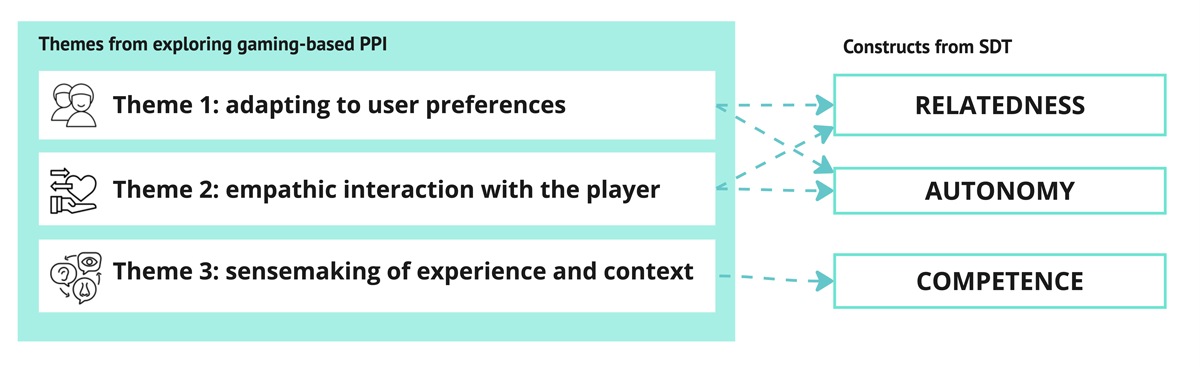
How the 3 themes correspond to basic psychological needs for relatedness, autonomy, and competence, in line with self-determination theory (SDT).

When revisiting the 3 themes and comparing them to related work, there appears to be a similarity between the identified findings of this study and the 3 basic psychological needs in SDT [103], which are: needs for autonomy, relatedness, and competence [92]; refer to Figure 8. As such, this study provides a form of bottom-up support for the usefulness of these constructs in designing and evaluating future gaming-based PPIs to understand how they might be more motivating to the user [92]. Further research should also investigate how and if a gaming-based PPI experience that does satisfy the relevant needs of autonomy, relatedness, and competence may contribute *in itself* to positive psychological outcomes for this population, as has been suggested [13].

Striking the right balance between learning and fun is a significant challenge, along with producing a relevant narrative [102] that supports intended learning. Ferrara [32] suggests a strategy for identifying the “gameness” that already exists in a context or situation rather than trying to *tack it on*. This may make it easier to transfer learning from the gaming space to everyday life [56,104]. However, moving from an idea to a game that works conceptually is challenging [105], and good intentions may be undermined by a seemingly fun yet unfit idea or concept, for example in the case of Disney and their first version of the game and exhibit “Habit Heroes,” intended to support healthy eating but rather reinforced stereotypes and made children feel bad about themselves [106]. Choosing an approach that “gamifies life” should thus be done with empathy, care, and frequent testing with users to avoid banalizing the situation and experiences of a vulnerable population, such as the young and unemployed. As such, a human-centered design approach is ideal because it starts with empathy [48]. However, frequent playtesting [56] and usability evaluation [57] are also needed to reduce the risk of developing a concept that is not engaging with the intended audience [88] or that undermines or does not foster learning. Established game genres and concepts could be used as inspiration in early explorative ideation. The characteristics of existing games may be viewed as opposing values on a spectrum [32], and by imagining what the game-based PPI would look like in the form of existing game genres, a large volume of different ideas can be formed that may be tested early for fit with the PPI objectives and user preferences.

**Figure 9:**
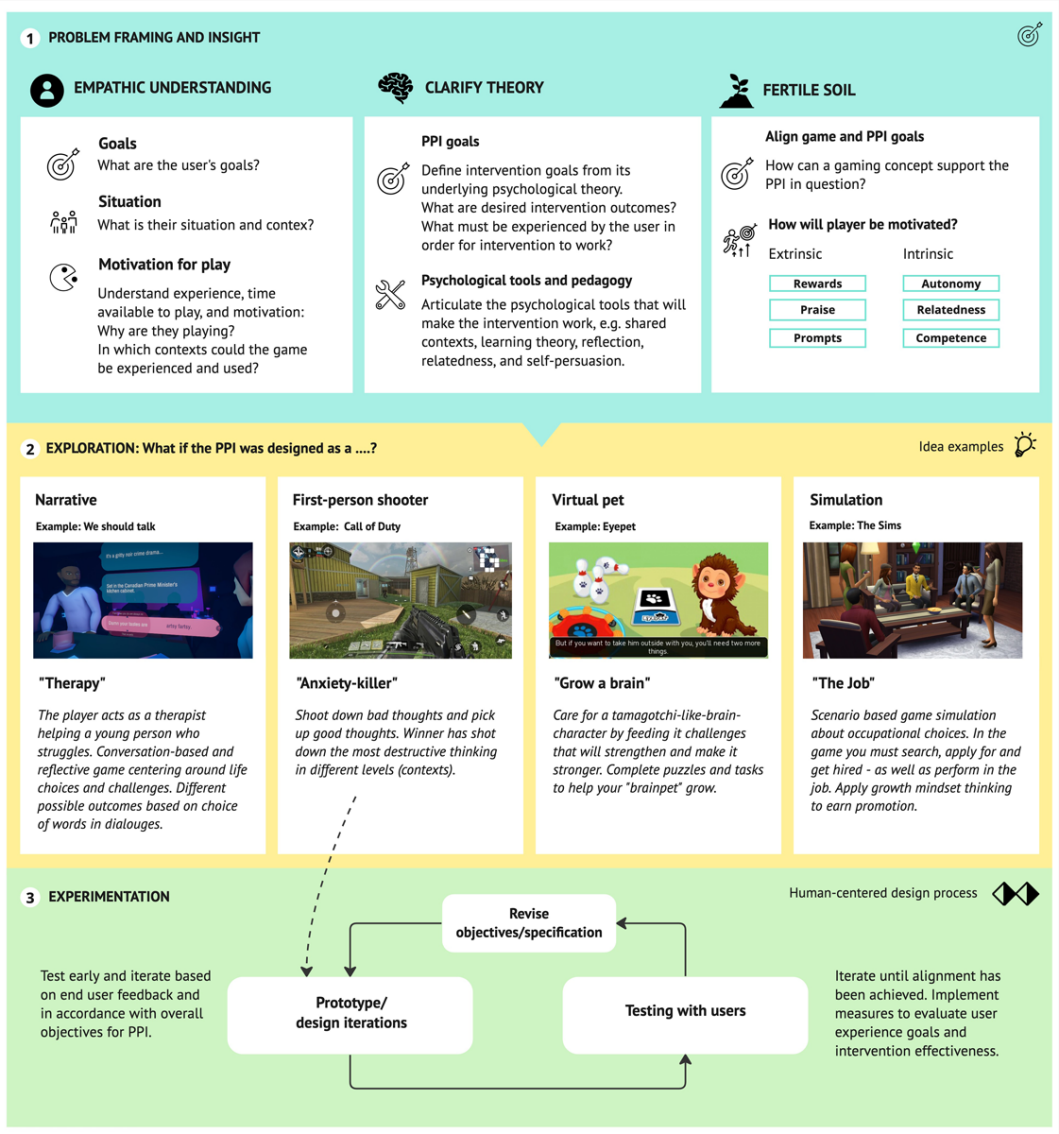
A proposed approach for designing future gaming-based positive psychology interventions (PPIs).

In Figure 9, we propose a broad but structured approach for how game-based PPI exploration may be executed, based on the lessons learned from our study and the discussion points in the preceding section. In this approach, insight into the user, context, gaming preferences, and gaming interests frames the design problem. It is also necessary to establish a clear and precise definition of PPI, including its underpinning mechanics, theories, and strategies that can help make the intervention effective. An alignment between the 2, forms the necessary “fertile soil” for the intervention game, where we ask how gameplay may support both user preferences and goals and PPI goals. Next, we propose working with existing genres and games to quickly generate many different ideas of what our gaming-based PPI may look and feel like. Promising concepts should be evaluated against relevant criteria, such as gameplay and user experience objectives and PPI objectives, and then made into prototypes for validation with user research.

The approach outlined here should be further detailed, refined, and validated in future research.

### Pointers for Future Research

There is a range of possible strategies to choose from to work to improve an intervention and increase user motivation. In this specific study and for the purposes of this paper, we explored one possible strategy: to attempt to make the PPI more engaging, user-friendly, and relevant for young, unemployed adults by creating a gaming-based intervention. There are other alternative strategies that could increase relevance, appeal, and adaptation to the needs of the target audience. Kelders et al [24] suggest the use of design and persuasive design techniques, including reward, praise and reminders [107] as a tool to increase motivation and retention. Others [13] suggest the use of SDT [97], as we also found some support for this study. These strategies should be explored further in future studies.

Furthermore, the alignment between gameplay and intervention does not rely solely on the crafting of the game. Although our study grounded ideas on learning from past empirical research where PPIs had been applied to different contexts, there was a lack of clarity and theoretical grounding for the user experience in itself, including a clear definition of the learning [35] that should happen within the game design space. Incorporating learning theory, such as experiential learning [41], along with motivation theory, such as SDT [97] and persuasive design [108], as a more complete theoretical framework for the game designing process may provide a stronger direction to the conceptual work for the practitioners involved. Designing for behavioral and mindset change is increasingly relevant for design research and professional design practice [109], and there seem to be several gaps in understanding for design researchers and design teams who find themselves grappling with psychological and behavioral theories to produce interventions to support problem-solving of societal problems, such as youth unemployment.

### Limitations

In this study, relevant participants were involved in a design process to capture their experience with designs in-the-making and take feedback into consideration in the design of revisions. We consider such early involvement a strength of the study. However, it also holds limitations; as the results are drawn from user experiences with *prototype* PPIs, the study does not provide user experiences resulting from a completed and verified PPI. Although the knowledge gained through the different stages of the design process is of substantial value to this area of research, future work is needed on experiences with fully functional gameplay PPIs to validate the findings of this study and to measure engagement, effectiveness, and adherence to the intervention. In addition, considering the fact that the positive psychologies implemented in the game mechanics were, to some extent, unclear to the participants after exposure, we cannot draw any conclusions on the experience of these in themselves at this stage. However, this was also not the purpose of this study.

Another limitation is the choice and availability of participants in the study. The population of unemployed young adults is highly heterogeneous. With our recruitment strategy, we are aware that we do not cover the entire range of end users, especially because we in part relied on voluntary registration and on contact with specific public welfare systems. Nevertheless, we find the involved participants to be within the scope of the studied PPI, and their feedback, hence, is of substantial benefit in understanding how the PPI may be experienced by representatives of this target group.

The third limitation concerns the context of the usability testing. Being observed by another person influences behavior (eg, Hawthorne effect), and participants likely spent much more time considering the prototypes than they would normally have. However, this approach was chosen because our interventions were prototypes and had unfinished functionality, which required a moderator to “fill the gaps” [110,111]. A fully self-administered and unmoderated use of a gameplay PPI would be a natural next step in future research.

Finally, it is important to note that, although our exploratory approach to insight into user perceptions of a game-based intervention for this target group is an important starting point for this area of investigation, future research is needed to establish the knowledge base needed to reliably provide such interventions. As part of this, we envision future studies with larger sample sizes and established scales as part of randomized controlled trials to gain further knowledge of the effectiveness of game-based interventions for this group and a basis for improvements in intervention design.

### Conclusions

The study contributes insights into key user perceptions of game-based interventions for unemployed young adults. The contribution has implications for future game-like intervention design for this purpose. Our principal contribution is to explore engagement through a PPI, designed as an interactive game. We have described the iterative process of the development of a 3D-game concept, VitaNova, and have explored participants’ thoughts and feedback on their experiences. Although the participants were positive about the general idea of a game targeted toward unemployed young people, we found tensions between a PPI and an exciting game play and 3 themes that pinpoint priorities for future gaming implementations. Our study shows that interactive game design could make interventions more entertaining and engaging but can easily come into conflict with or undermine the intervention. We recommend aligning the gameplay narrative, objectives, and mechanics with intervention content and objectives to create engaging, relevant, and effective gaming-based PPIs that promote a more productive view of the challenges experienced by the young and unemployed.

## Appendix

Multimedia Appendix 1 A presentation of the different game prototypes.

## Abbreviations

CV: curriculum vitae
IPS: Individual Placement and Support
NAV: Norwegian Labour and Welfare Administration
NEET: not in education, employment, or training
PPI: positive psychology intervention
SDT: self-determination theory
