# Supplementary material for: Exploring a Gaming-Based Intervention for Unemployed Young Adults: Thematic Analysis
Source: JMIR Hum Factors. 2024 Jan 18;11:e44423. doi: 10.2196/44423 (PMC10835587; doi:10.2196/44423)
Supplement: Multimedia Appendix 1 [file humanfactors_v11i1e44423_app1.pdf]

Universitetet i Stavanger

31. Aug 2023

Presenting the game prototypes

Appendix to paper

Steps	1: Exploration	2: Agile development	3: Evaluation
Main design activities	Design sprint and MVP testing.	Agile development and design.	Refine and adjust. Evaluate.

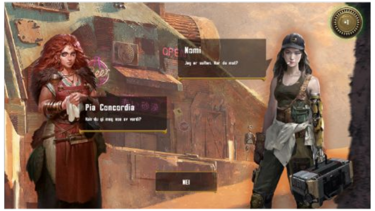

VitaNova v1

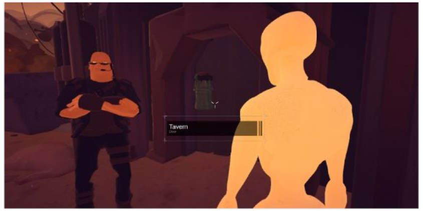

VitaNova v2

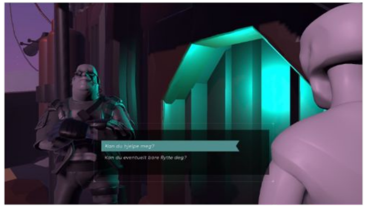

VitaNova v3

Different iterations during design process

v1 MVP from Google Design Sprint

A 2D-version of the game made after a Google design sprint.

Include psychological content embedded in external videos that you need in order to answer in-game quizzes and more.

You accept a challenge to run a taverna and have to hire people to help you.

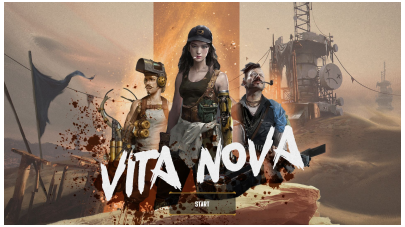

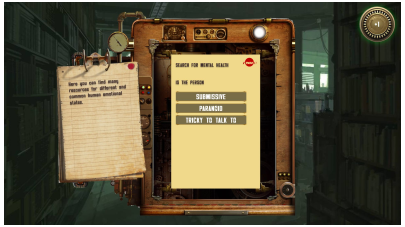

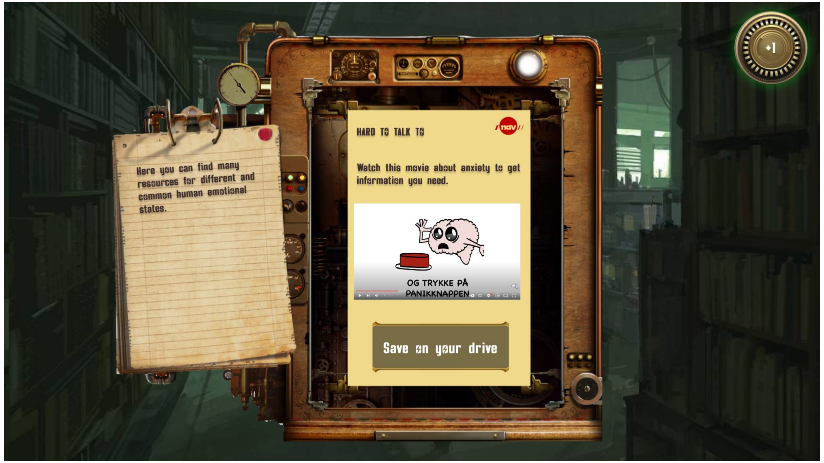

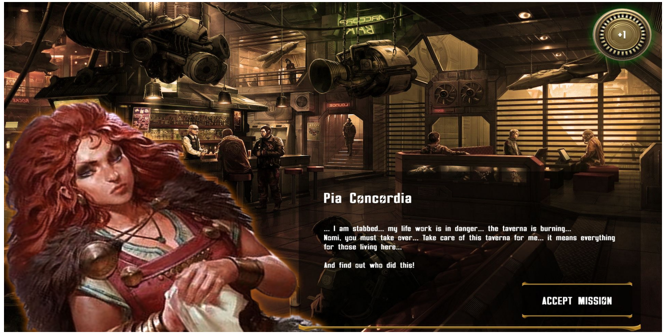

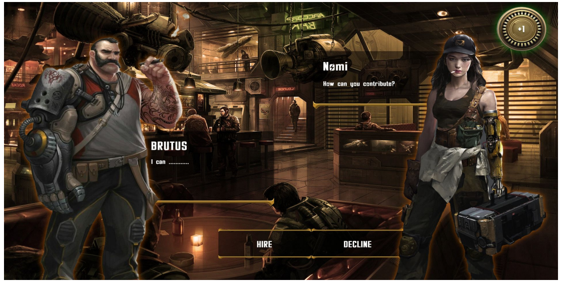

v2 agile development prototype

A 3D version of the game, now built in Unity web GL.

This version of the prototype includes some open world-like features, such as a possibility to look freely around to explore. We experience some technical problems with the loading time of the game.

New features are added such as possibility to pick up items in the 3D environment to add to your inventory, things you can to solve tasks such as hacking door lock.

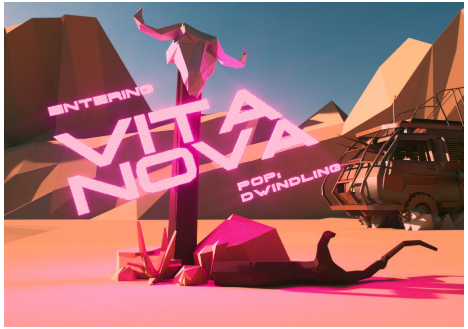

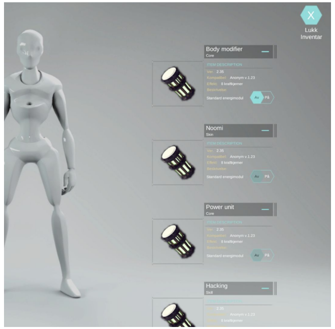

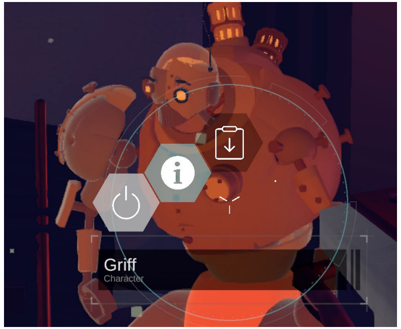

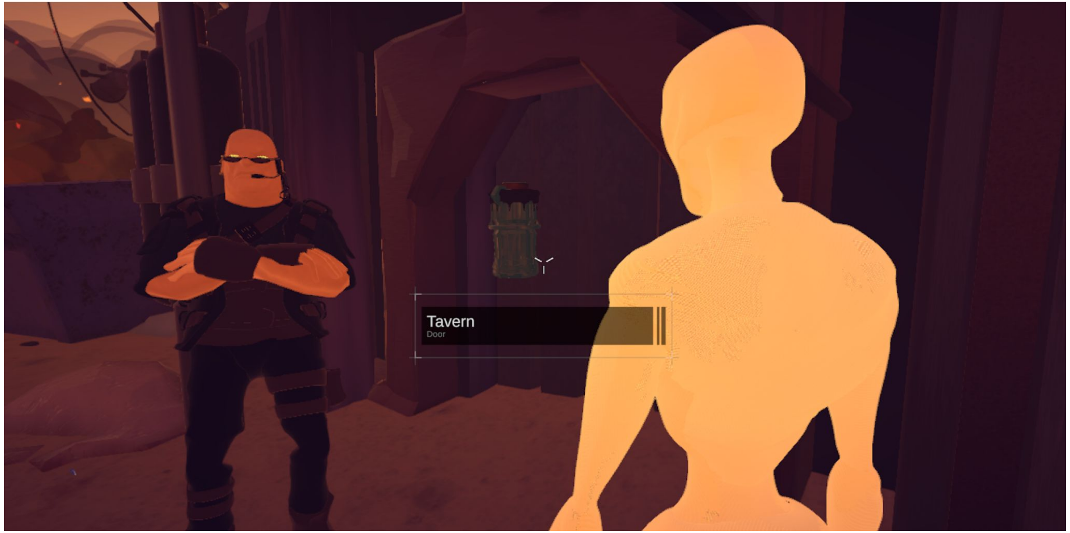

v3 refinement

Improved graphics and more of the content from the USAY/NSLM study content on growth mindset added into the game narrative, for instance embed into game the metaphor of the brain as a muscle.

Division of game into episodes, to enable use over 8 weeks with one weekly episode.

Added reflective questions at the end of each episode.

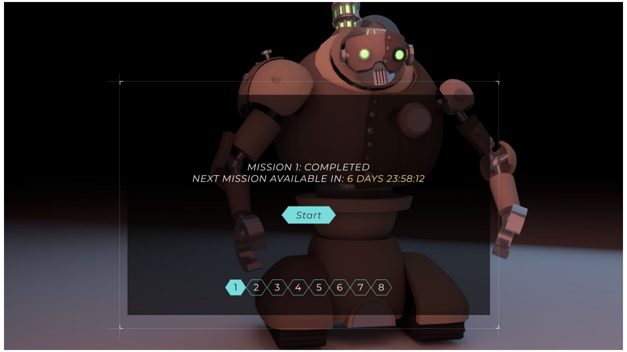

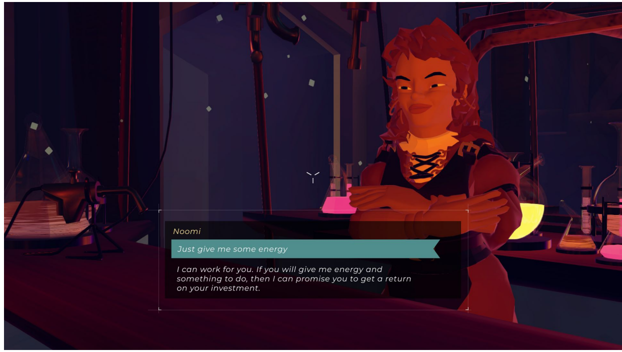

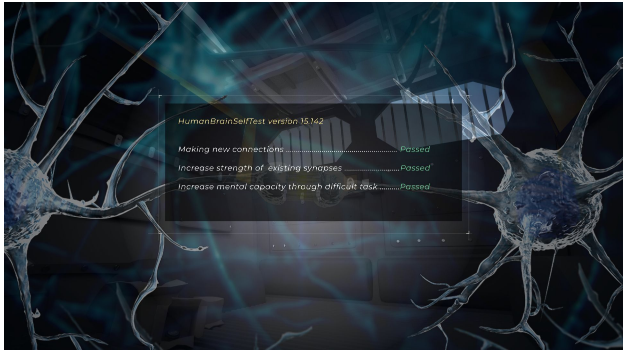

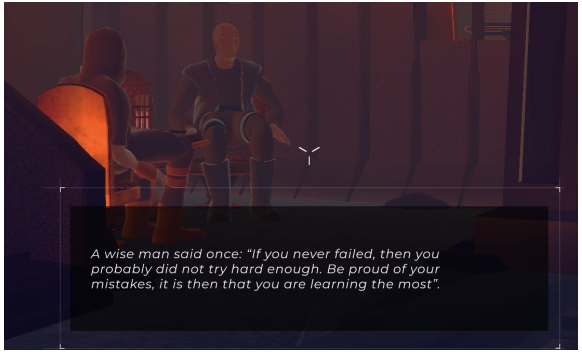

Questions or queries?

ingjerd.j.straand@uis.no
